# A Clinical Lecture Delivered at the Bellevue Hospital, New York

**Published:** 1879-12

**Authors:** Austin Flint

**Affiliations:** Professor of the Principles and Practice of Medicine in the Bellevue Hospital Medical College


### A CLINICAL LECTURE DELIVERED AT THE BELLEVUE HOSPITAL, NEW YORK.

By AUSTIN FLINT, M.D..
Professor of the Principles and Practice of Medicine in the Bellevue Hospital Medical College.

*Gentlemen*—I shall introduce two patients having valvular lesions of the heart, and, after stating the important clinical facts which the cases illustrate, I shall ask your attention to some practical remarks on valvular lesions.

This man's name is Andrew R. He is 47 years of age, and he is a blacksmith. His family history is unimportant. Prior to his present illness he always had good health. He was a strong, robust man. He has never had syphilis, nor rheumatism, nor any important pulmonary disease. He has been a moderate drinker. Six years ago he began to suffer, at times, from dizziness and palpitation. Coincident with these symptoms he passed blood with the urine. From that time to this, he has had repeatedly recurring attacks of hæmaturia, the amount of hemorrhage being sometimes considerable, and attended with obstruction to the free passage of urine. The attacks have been of short duration, and, in the intervals, he has been free from any urinary difficulty. The last attack was three weeks ago, just before his admission into hospital. He has an anæmic facies, and there is some œdema of the lower limbs and face. The apex beat of the heart is feeble, and felt in the fifth intercostal space just within the *linea mammalis*. There is a mitral systolic murmur, not transmitted much to the left of the apex. A systolic murmur is heard at the base, and the same murmur is heard over the carotids. There is also a feeble aortic regurgitant murmur. The aortic second sound is not weak, but the pulmonic second sound has greater intensity. The area of præcordial dullness on percussion is slightly increased.

I shall not consider the pathological significance of the hæmaturia in this case, except to say that it is true hæmaturia and not hæmatinuria, for the blood coagulates after its emission; that the long period since the first attack occurred renders the existence of malignant disease improbable, and that examinations of the urine now give no evidence of disease of either the bladder or the kidneys. I will add that one of my surgical colleagues, who has explored the bladder, is led to suspect papilloma within that organ. The hemorrhages have doubtless contributed to the anæmia.

I desire to call attention especially to the heart. We have the evidence of valvular lesions, both mitral and aortic. Moreover, there is some cardiac enlargement. Now what has the heart to do with the dropsy and the general condition of this patient? Very little, if anything. It would be a mistake to treat the patient as if the cardiac affection were the objective point, and prescribe digitalis, which, by the way, I apprehend is too often prescribed, as a matter of course, if there be any disease of the heart. It would be a mistake to give diuretics or hydrogogues to get rid of the dropsy, which is not enough to occasion any inconvenience. The desirable end is the building up of the patient. He needs good alimentation, digestion, assimilation, and nutrition. If we can succeed in giving to this patient good blood and improving his general health, I venture to predict that, for the present at least, his cardiac lesions will give him little or no trouble.

The name of the other patient is Mary A. C. She is 45 years of age. She is married, and has two healthy children. Her parents are both dead, but she does not know of what diseases they died.

Fifteen years ago she had acute articular rheumatism, and she had another attack six months ago. Since the last attack she has suffered from dyspnœa, with cough, some pain in the præcordial region, and a sense of constriction in the chest. Her appearance is anæmic. She suffers from dyspnœa on any exertion, and is sometimes obliged to get up at night. The apex-beat of the heart is in the sixth intercostal space two inches without the *linea mammalis.* The præcordial dullness is considerably extended, and increased in degree. The impulse of the heart is weak. At the apex there is a presystolic together with a systolic murmur, the latter transmitted into the axillary region and heard on the back. There is also a systolic murmer at the base, and this is heard over the carotids. The pulmonic is notably louder than the aortic second sound.

We have in this case mitral and aortic valvular lesions with considerable enlargement of the heart. General dropsy is wanting; but the patient has grave and, of course, incurable cardiac disease. Now, aside from this disease, she is anæmic and feeble. Perhaps her general condition can be improved by tonic remedies, good alimentation, etc. If this can be done, she may suffer comparatively little from the cardiac disease. The important object of treatment in this case is to improve to the utmost the general condition, in order thereby to secure the best possible tolerance of a disease which must necessarily continue.

Taking these two cases as a text, I shall devote the remainder of this lecture to some considerations relating to the diagnosis and treatment of valvular lesions.

The recognition of valvular lesions and their localization at one or more of the valves or orifices of the heart, have been brought to such precision by the study of the cardiac murmurs, as apparently to leave nothing to be desired further in these points of view. Important as are the advantages of this precision of physical diagnosis, it has not been without some disadvantages in relation to prognosis and treatment. Let me point out certain evils which may proceed from an accurate knowledge of the existence and seat of valvular lesions.

Nearly twenty years ago I was consulted by a physician from one of the Southern States. He had recently graduated in Philadelphia. Before leaving Philadelphia, he was led to ask the late Dr. Gerhard to examine his chest, some of his relatives having died with phthisis. Dr. Gerhard pronounced his chest free from any signs of pulmonary disease, but told him that he had a heart murmur. Prior to this time, he had never had any symptoms pointing to cardiac trouble. The idea of the murmur worried him, and directed his attention to the heart. Some time afterward he was conscious of a little disturbance of the heart's action, and he went to Philadelphia for another physical examination. He was then examined by the late Dr. Pepper, who told him that there was a murmur, but no other evidence of any affection of the heart. He was comforted by Dr. Pepper's assurances, but was unable to dispel altogether his apprehensions. Under these circumstances, he requested me to examine the heart. I found a systolic murmur within a limited area at the apex. The heart was not enlarged. Its action was regular. The pulmonic and aortic valvular sounds were normal. I assured him that there was no present occasion for anxiety, and advised him to try to forget that he had a cardiac murmur. He adopted my advice. He has ever since been engaged actively in medical practice. No symptoms of disease of the heart have occured. During the last summer he called upon me, and I examined the heart. I failed to discover any murmur, and in all respects the organ was normal.

I cite this case as a striking one, among a considerable number of analogous cases, teaching the same lesson. The lesson is, that a cardiac murmur undoubtedly organic, that is, not hæmic nor extra cardiac, may represent a lesion which, for the nonce, is innocuous, and may never become otherwise. Now, what evil consequences may follow the discovery of such a murmur? A patient is told that there is the evidence of something wrong in the heart. In common parlance, he has "heart disease." In the popular mind this means always something grave, and is generally considered as involving more or less danger of sudden death. Let it be supposed that the physician enjoins extreme care in regard to physical exercise and mental excitement, advising perhaps the relinquishment of the pursuit in which the patient is engaged; impressing the importance of complete inactivity. It can easily be imagined how much anxiety is produced, and that the duties and aims of life may be sacrificed to needless precautions. These consequences are not imaginary; they have been exemplified in not a few instances which have fallen under my observation. In the instances referred to, it would have been better for the patients had a stethoscope never been applied to the chest. So great in some instances have been the evils following the discovery of an organic murmur, that ignorance of its existence would have been comparatively blissful.

How are such evils to be avoided? By acting in accordance with the following wholesome medical maxims: Positive, proximate evils are not to be incured in view of remote, doubtful contingencies. A patient is always entitled to a prognosis as encouraging as is consisted with the most hopeful aspect of the case. I will endeavor to illustrate the application of these maxims by a hypothetical case. A physician examines the chest of a patient who has no symptoms of cardiac disease; following the rule that the examination should always enter into a thorough investigation of a case, or perhaps, because the patient wishes to be assured that he is sound, a cardiac murmur is recognized, and the conclusion is reached that it is not an inorganic murmur. The organ is not enlarged. The impulse and the characters of the first sound that its muscular power is normal. The aortic and the pulmonic second sound have a proper relative intensity. Under these circumstances, the murmur represents a lesion of some kind, it is true, but one which, for the time being is innocuous. The lesion may, or may not, at a future time, lead to hypertrophy and dilatation. These results, if they ensue, will probably give no manifestations until after the lapse of many years. Acting in accordance with the maxiums just stated, the physician need not volunteer any statement respecting murmur. If the question whether there be anything wrong be asked, a truthful answer must, of course, be given; but the statement should be accompanied by such explanations and assurances as will preclude the idea of the existence of any important disease, or the expectation of its development in the future. The patient is to be spared apprehensions which, of course, are gratuitous if the lesion remains permanently innocuous. Moreover, if hypertrophy and dilatation follow, at a period more or less remote, apprehensions will not prevent these results, and may even tend to promote them. And, in this connection, let me state a fact, already implied, which underlies the prognosis and treatment whenever the existence of valvular lesions is ascertained. The remote pathological results proceed, not directly from the valvular lesions, but from the hypertrophy and dilatation which they induce, and chiefly from the latter of these two varieties of enlargement of the heart.

In the case which I sketched of the physician, a mitral systolic murmur represented an innocuous lesion. The murmur in that case was not a regurgitant murmur, although systolic. Here is an important distinction which may often be made clinically. A systolic murmur, having its maximum of intensity near the apex, propagated a greater or less distance in a lateral direction to the left, and heard on the back near the lower angle of the scapula, is evidence of mitral insufficiency. But, if limited to a space at the apex, it has not that significance ; it may be due to an intra-ventricular lesion, the competency of the valve not being impaired, and, consequently, no regurgitant current taking place. My colleague, Prof. Janeway, has demonstrated that a small tendinous cord attached to opposite points of the inner walls of the left ventricle—a congenital abnormity—may cause an intra-ventricular systolic murmur. But a truly regurgitant mitral murmur does not necessarily represent a lesion of present importance. An insufficiency so small as to allow a regurgitant stream no larger than a knitting needle, may give rise to a murmur, loud, propagated to the left, and heard on the back. Such a lesion occasions no appreciable disturbance, and it may never increase. Practically it is, and may remain indefinitely, innocuous. And here let me remark that a practical precept is never to draw any inference concerning the amount of lesion from the intensity of a murmur. It is perhaps true, as a rule, that the smaller the amount of regurgitation, the louder the regurgitant murmur.

What is true of mitral lesions is equally true of those seated at the other orifices. An aortic direct murmur which, from its persistence and other circumstances, is not of hæmic origin, whether feeble or intense, may represent only a patch of atheroma or calcification causing no pathological effects, and remaining innocuous indefinitely. A little insufficiency of the aortic valve may have no greater importance than that of a mitral lesion causing a small regurgitant current. That an aortic regurgitant murmur may persist many years without enlargement of the heart, and giving rise to no appreciable cardiac symptoms, I know from cases which have been for a long time under my observation. As much can be said sometimes of a mitral direct or obstructive murmur ; but I shall presently speak of the remarkable tolerance of the lesions giving rise to this murmur.

The practical points which I wish to make are these : Valvular lesions represented by murmurs, if there be no enlargement of the heart, are not of immediate pathological importance, and they may never become important. If the knowledge of their existence can be properly withheld from patients, it had better not be communicated. By no means terrorize them with the idea that they are doomed to die of "heart disease." Do not enjoin any radical changes in life, provided their habits are not inconsistent with hygienic laws. Above all, forbear any efforts to remove the lesions by mercury, the iodide of potassium, or other remedies. This last injunction may seem to many superfluous: yet it has happened that within a few days I have received a letter from a medical practitioner detailing a case of a valvular murmur with no enlargement, the object of the letter being to inquire as to the propriety of other treatment than by large doses of the iodide of potassium which the patient was taking. In short, reverting to the maxims which I have laid down, avoid destroying the happiness and usefulness of a life in view of possible remote contingencies; allow all the benefit of a prognosis based on the fact that an innocuous lesion may remain so indefinitely, and may never become otherwise.

I proceed next to offer remarks on valvular lesions which have led to more or less enlargement of the heart. Errors in relation to prognosis and treatment may arise, in the first place, from attributing cardiac symptoms dependent on a functional affection associated with, but not dependent on, lesions, wholly to the latter; in the second place, from a deficient appreciation of other morbid conditions in accidental association, to which are measurably due the supposed effects of the cardiac lesions; and, in the third place, from an under-estimate of the ability to tolerate these lesions.

It happens not infrequently that a patient with valvular lesions and more or less enlargement, who experiences little or no inconvenience therefrom, becomes affected with functional disorder from the same causes which produce it when the heart is sound, namely, dyspepsia, anæmia, the abuse of tobacco, excesses in venery, and prolonged mental worry. The functional disorder is independent of the organic affections, although, doubtless, more distressing in proportion as the heart is damaged by the latter. Now the palpitations, irregularity of rythm, and intermissions, in some cases so excessive as to render significant Bouillaud's expression, *folie de cœur*, may be attributed wholly to the organic affections; hence, an exaggerated estimate of the importance or gravity of these. The physician may be taken by surprise, and astonished by the disappearance of symptoms which seemed to be alarming, and an apparent recovery of the patient. Many years ago I saw in consultation a young man who had unmistakably a valvular lesion, and there was such an amount of disturbance of the heart's action that a most unfavorable prognosis was given. Some months afterward the attending physician sent him to report to me. He considered himself well, and was actively engaged in some mechanical employment. He had a lesion which gave him no inconvenience, and the symptoms on which were based the unfavorable prognosis were functional, not dependent on the organic affection. I am free to say that I could cite other analogous instances of error in prognosis from my own experience.

If valvular lesions be associated with little or no enlargement, co-existing disturbances of the heart's action are to be considered as functional, and especially if the causative agencies of the latter be discoverable. But when the associated enlargement is considerable, it is by no means always easy to determine to what extent the cardiac symptoms are due to a functional disorder which is independent of the organic affections. This point is often only to be settled by the removal of existing causes of functional disorder, and appropriate treatment in other respects. A large clinical experience is of much use in forming a judgment on this point, before it is settled by time. As bearing thereon, the persistence and the effects of a purely functional disorder, that is, not associated with any lesion, in some cases, are to be considered. The following is a sketch of a striking illustration which came under my observation about two years ago. The patient, a lady in the neighborhood of forty years of age, had long suffered from dyspeptic ailments, and frequently recurring attacks of functional disorder of the heart. At the time to which I refer, the organ was in a state of persistent palpitation for several successive weeks. The pulse was thready, and extremely frequent, often exceeding 150 per minute. She became anasarcous, and the face had the pallor of death. It was difficult to realize that she was not in danger of dying at any moment. Yet, repeated examinations prior to this attack had shown that there was no enlargement, and there were no signs of valvular lesion. At length the functional disorder ceased, and she has since had comfortable health, although suffering at times from dyspepsia and paroxysms of palpitation.

By morbid conditions, other than functional disorder of the heart, accidentally associated with cardiac lesions, which are liable to give rise to error in diagnosis and treatment, I do not mean complicating affections as, for example, Bright's disease. The conditions especially referred to are impoverished blood, impaired nutrition, and general debility. A patient with obstructive or regurgitant lesions, or both combined, and the heart more or less hypertrophied, may scarcely be aware of any cardiac affection so long as alimentation is abundant, the digestion good, assimilation unaffected, the body well nourished, the nervous system undisturbed, and the muscular strength well maintained. Now, let it be supposed that this patient, from loss of appetite or any cause, ingests an insufficient supply of food; or that the digestive functions are impaired; or that either the assimilatory or digestive processes are defective; or that nervous prostration is induced by overwork or other causes; or that the muscular system is exhausted. Under these circumstances, singly or combined, distressing symptoms or effects of the cardiac affection are manifested. Dyspnœa and general dropsy, if the seat of the lesions be mitral, and præcordial oppression if the seat be aortic, are more or less marked. The error in prognosis is the belief that these symptoms and effects represent purely and simply the cardiac lesions, whereas they are measurably caused by co-operating morbid conditions accidentally associated therewith. The error in treatment is in directing measures exclusively to the heart. If the associated morbid conditions can be removed, the patient is restored to the comfortable state which existed prior to their occurrence. The object of treatment is the removal of these conditions.

Illustrations of the point which I am endeavoring to make clear, are frequent both in private and in hospital practice, more especially the latter. Over and over again I have presented in this amphitheatre, patients suffering from dyspnœa and general dropsy, and ventured to predict that, by appropriate treatment addressed not directly to the heart, but to associated morbid conditions, so much improvement might be hoped for that it would seem as if recovery from the cardiac affection had taken place. I could cite numerous cases in which this prediction was fulfilled. The grounds for such a prognosis are the existence of some, or perhaps all, of the conditions which have been named, and the degree of hypertrophic enlargement of the heart not being sufficient alone to account for the symptoms and effects.

I cannot leave this topic without commending it to your thoughtful consideration. Its importance as bearing on prognosis and treatment in cases of valvular lesions with enlargement of the heart, is very great.

Errors, in relation to prognosis and treatment, arising from an under estimate of the tolerance of cardiac lesions, are common, as a consequence of limited clinical experience. Giving preference to my own errors, over those of others, I will cite the following illustration: A family to which, nearly thirty years ago, I stood in the relation of physician, consisted of a husband, a wife, and three daughters. All were healthy except one daughter, about fourteen years of age, who was attending school, and appeared less robust than her sisters, without being considered an invalid. On making an examination of her chest I was surprised to find a loud mitral regurgitant murmur, and considerable hypertrophic enlargement of the heart. Disease of the heart had not been suspected prior to this examination. I felt it my duty to tell the parents that they must not expect her to reach maturity, and that, probably, at farthest, she would live but a few years. Fortunately, perhaps, for her, they attached not much importance to my communication. She continued to go to school, and no restrictions of any kind were placed upon her. The girl developed into a delicate looking woman, but with comfortable health, dying with the usual symptoms of mitral lesions and dilatation of the heart, about twenty years after the date of my examination, surviving her father, mother, and one of her sisters. The error in this case was not in the diagnosis, but in the prognosis.

If valvular lesions with enlargement be unaccompanied by associated unfavorable morbid conditions, they are often well tolerated for a long period. To promote tolerance, the object of treatment is to maintain a maximum general health. It is a great mistake to impoverish the blood, impair nutrition, and induce general debility by a restricted diet, and to enjoin complete mental and physical inactivity. A diet abundant and nutritious is a *sine qua non*. Restrictions should be determined only by the digestive capabilities. Muscular exercise, within a certain limit, is advisable from its influence on appetite, digestion and nutrition. The proper limit is easily defined. It should not be so active as to occasion discomfort from either the action of the heart or dyspnœa. It should not be continued so as to produce exhaustion. Mental occupation, within the bounds of comfortable endurance, is useful as a hygienic measure, and as a preventive against introspection and brooding. It is, perhaps, not incorrect to say that practical errors in relation to the foregoing points in the treatment are not rare.

The situation and the character of valvular lesions are to be taken into account in prognosis and treatment. Aortic lesions which allow free regurgitation are the most doubtful in the way of prognosis. These lesions especially involve the liability to sudden death. They interfere with the nutrition of the left ventricle, inasmuch as the passage of blood into the coronary arteries takes place in the diastole, being effected by the recoil lessened in proportion as the aortic valves are insufficient. The danger of sudden death is greater without, than with, mitral insufficiency. The latter is to a certain extent protective against accumulation of blood in the left ventricle, and paralysis from distention. Weakness of the left ventricle from dilatation augments greatly the danger. These are points to be considered in the prognosis. As regards treatment, the object is to maintain the hypertrophy and muscular vigor of the left ventricle. Digitalis, given to the extent of diminishing the frequency of the heart's action, or, in other words, lengthening the pause after the two sounds, may increase the danger from over-filling of the ventricle.

Aortic obstruction is well tolerated. It is surprising how contracted the aortic orifice sometimes becomes before death takes place. This is better borne with, than without, mitral insufficiency. Regurgitation into the left auricle is compensatory and conservative.

Mitral regurgitant lesions, which are the most frequent in occurrence, may be tolerated indefinitely under favorable accompanying conditions. The case which I last cited exemplifies this statement.

Of mitral obstructive lesions I will speak at somewhat greater length. The murmur which represents these, namely, the mitral obstructive, or direct, or presystolic, has been recognized only within a few years. It was formerly confounded with the mitral regurgitant murmur, and it is still by some who have given more or less attention to asculation as applied to diseases of the heart. Not one of the cardiac murmurs is more readily recognized than this, when in its distinctive characters are clearly apprehended. No other murmur occurs after the second sound and before the first sound, ending with the latter. Moreover, generally it has a peculiar vibratory or blubbering character, and it is limited to a circumscribed space around the apex of the heart.

This murmur represents mitral obstructive lesions, with an important exception, namely, when there is free aortic obstruction. I may claim priority in pointing out this exception, and in offering an adequate explanation of it. For the latter, I must refer you to my work on "Auscultation." I may state here that this murmur is by no means so rare as I supposed when my clinical experience was more limited than at the present time. And it occurs oftener without mitral regurgitation than I formerly supposed. I have lately observed an interesting fact in regard to this murmur, namely, it may disappear after mitral obstruction has led to great dilation of the left auricle. Under these circumstances, at different times, it may be present and absent. Its disappearance, of course, is of unfavorable omen, preceding or accompanying increase of dyspnœa and of general dropsy. When I first studied this murmur, I had the impression. that it very rarely existed without a co-existing mitral regurgitant murmur. Experience has shown me that this impression was erroneous. In the majority of cases mitral obstruction is combined with mitral insufficiency, but the instances of the former without the latter are not infrequent.

Mitral obstruction, as represented by the presystolic murmur, may be tolerated indefinitely, and is not incompatible with comfortable health. Of a pretty large number of cases sustaining this statement, I will refer to a few.

Twelve years ago I saw a woman a few days after her confinement. She had orthopnœa, cyanosis, much anasarca, and a large dropsical effusion into both pleural cavities. There was a mitral direct murmur, with moderate enlargement of the heart. The physician who attended her in labor said the dyspnœa was such as to lead him to fear that she would die before the birth of her child. She apparently regained good health. Meanwhile, I have seen her once, in consultation, with a moderate general dropsy. This was several years since, and I have lately heard of her as in comfortable health.

I have notes of two cases, both women, who were under my observation for more than ten years. In both the mitral obstruction did not interfere with comfortable health. In one case death at length took place, preceded by much suffering from dyspnœa and by general dropsy. In the other case, while the patient continued to suffer but little, and was accustomed to much activity, death was caused by gangrene of both lower limbs, probably due to embolism.

In another case which has been under my observation for ten years, the patient suffers but little inconvenience from symptoms referable to the heart, although the general health has been much impaired at times by malarial fever and by menorrhagia. In this case the mitral direct is associated with an aortic regurgitant murbur. During the ten years, very little, if any, enlargement of the heart has taken place.

I will next offer some remarks on the treatment in cases of mitral lesions (obstructive or regurgitant, or both combined) associated with dilation of the heart, sufficient, exclusive of accessory morbid conditions, to give rise to dyspnœa, orthopnœa, and to general dropsy. Every practitioner of much experience is familiar with cases in which the patient is unable to lie down on account of distress from the sense of the want of breath, getting a certain amount of disturbed sleep by bending the body forward, raising the arms, and resting the head upon some support. There is anasarca, and the lower limbs, together with the scrotum and penis, become enormously swollen. There is more or less effusion within the peritoneal cavity and both pleural cavities. The suffering for days, weeks and months in this pitiable condition, is extreme. Death seldom comes in a shape more terrible than this. What can be done to diminish suffering and prolong life under these circumstances?

Efficient measures of treatment will often do much. The effect of active hydragogue purgation is sometimes marvelous. Physicians are often unduly timid in resorting to potential hydragogues for fear of exhaustion. The elimination of water by the intestinal canal, so far from exhausting the patient, gives increased strength by relieving the dropsy and the suffering from dyspnœa, thereby enabling the patient to have refreshing sleep. Elaterium, the king of hydragogues, may be judiciously given without apprehension. Podophyllin is a royal remedy, but less potent than elaterium. Of the inferior grade of hydragogues, the pulvis purgans should hold a high rank; but in my experience it has often proved unreliable, as I suspect from the poor quality of jalap dispensed by apothocaries. The Epsom salts is an old remedy which has fallen in a measure into unmerited disuse. It is easily administered, generally well borne and reliable. After relief has been produced by active hydragogue purgation, either a moderate drain from the bowels may be kept up, or the active purgation repeated once a week or fortnight, as circumstances may indicate. In the intervals, a diet as substantial as practicable, and tonic remedies, if indicated, are important, adopting the excellent maxim that "a lame heart needs good blood."

In the cases to which I now refer, digitalis is often an invaluable remedy. This remedy is indicated by weakness, rapidity and irregularity of the heart's action. I agree with those who regard this remedy as a cardiac tonic rather than a cardiac sedative. Its potency is sometimes extraordinary. It should be given for a short time, and then suspended, to be resumed, from time to time, according to the indications.

I will conclude this lecture by summarizing in a few aphorisms the practical points which I have presented.

1. Cardiac murmurs may represent lesions which, if unaccompanied by symptoms referable thereto, enlargement of the heart not coexisting, and the heart-sounds normal, are to be considered as innocuous. The prediction of grave consequences, under these circumstances, is unwarrantable, inasmuch as they may never occur. Such lesions do not claim medical treatment, nor any extraordinary precautions; and it is desirable that the fact of their existence be withheld from patients, if this can be done with propriety.

2. Patients with valvular lesions are liable to suffer from functional disorders of the heart, arising from causes which have no pathological connection with the lesions. It is highly important to recognize, clinically, this accidental coincidence, in order to exercise a correct judgment as to the prognosis and treatment.

3. Various morbid conditions, other than functional disorder of the heart, may be accidentally associated with valvular lesions and more or less cardiac enlargement. These associated morbid conditions may be, in a great measure, responsible for symptoms and effects which seem to denote an advanced stage of the cardiac disease, whereas, the latter may occasion but little inconvenience, provided these accessory, co-operating conditions can be removed.

4. Valvular lesions involving either obstruction or regurgitation, or both combined, and having led to considerable or even great enlargement of the heart, under favorable circumstances as regards associated morbid conditions, are often well tolerated indefinitely. There is less reason for a hopeful prognosis, in respect of tolerance, when there is considerable aortic insufficiency, than in cases of aortic obstructive lesions, and those which occasion obstruction or regurgitation at the mitral orifice. The danger of sudden death from aortic regurgitation is lessened by co-existing mitral insufficiency.

5. In cases of orthopnœa and general dropsy dependent on mitral obstructive or regurgitant lesions and enlargement of the heart, digitalis and active hydrogogue purgation repeated from time to time, not only often afford notable relief, but there is reason to believe that life is sometimes thereby much prolonged.

